# Prospects for the Study and Improvement of Abiotic Stress Tolerance in Date Palms in the Post-genomics Era

**DOI:** 10.3389/fpls.2020.00293

**Published:** 2020-03-17

**Authors:** Khaled Michel Hazzouri, Jonathan M. Flowers, David Nelson, Alain Lemansour, Khaled Masmoudi, Khaled M. A. Amiri

**Affiliations:** ^1^Khalifa Center for Genetic Engineering and Biotechnology, United Arab Emirates University, Al Ain, United Arab Emirates; ^2^Center for Genomics and Systems Biology (CGSB), New York University Abu Dhabi, Abu Dhabi, United Arab Emirates; ^3^Center for Genomics and Systems Biology, New York University, New York, NY, United States; ^4^Date Palm France Biotechnology, Châteauvieux, France; ^5^College of Food and Agriculture, Department of Integrative Agriculture, United Arab Emirates University, Al Ain, United Arab Emirates; ^6^College of Science, Department of Biology, United Arab Emirates University, Al Ain, United Arab Emirates

**Keywords:** date palm, abiotic stress, omics, desert microbiome, genetic transformation, breeding program

## Abstract

Date palm (*Phoenix dactylifera* L.) is a socio-economically important crop in the Middle East and North Africa and a major contributor to food security in arid regions of the world. *P. dactylifera* is both drought and salt tolerant, but recent water shortages and increases in groundwater and soil salinity have threatened the continued productivity of the crop. Recent studies of date palm have begun to elucidate the physiological mechanisms of abiotic stress tolerance and the genes and biochemical pathways that control the response to these stresses. Here we review recent studies on tolerance of date palm to salinity and drought stress, the role of the soil and root microbiomes in abiotic stress tolerance, and highlight recent findings of omic-type studies. We present a perspective on future research of abiotic stress in date palm that includes improving existing genome resources, application of genetic mapping to determine the genetic basis of variation in tolerances among cultivars, and adoption of gene-editing technologies to the study of abiotic stress in date palms. Development of necessary resources and application of the proposed methods will provide a foundation for future breeders and genetic engineers aiming to develop more stress-tolerant cultivars of date palm.

## Introduction

Among the greatest challenges currently facing crop productivity worldwide are the salinization of arable land and pressures from various sources of abiotic stress. These challenges are exacerbated in arid and semi-arid regions where climate change and chronic water shortages have reduced arable land area and reduced crop yields. As fresh water resources are depleted, irrigation with brackish water, drought, evaporation and excessive heat in these regions cause large amounts of soluble salt to accumulate in the soil. These conditions, together with other sources of abiotic stress such as heavy metal exposure and nutrient stress, increasingly pose a threat to crop yields and food security.

The date palm (*Phoenix dactylifera* L., 2*n* = 36) is a commercially important fruit crop in arid regions of the Middle East and North Africa. Date palms are dioecious and have a long juvenile phase that includes a minimum of 4 years to first flowering and 10 years or more to produce basal offshoots and reach maximum yield capacity (Chao and Krueger, 2007). Like other dioecious fruit crops, cultivation practices utilize vegetative propagation such as transfer of basal offshoots or micropropagation in tissue culture to clone female cultivars. These practices maintain a rich diversity of more than 3,000 named varieties worldwide which are valued primarily for their sweet fleshy fruits (Zaid and de Wet, 2002). Micropropagation has become the primary means of propagating elite cultivars for commercial production in many areas, but bringing new cultivars into tissue culture is difficult and time consuming (Aaouine, 2003; Mazri and Meziani, 2015). Sexual reproduction has also been adopted in some areas, but seedlings are undesirable in most commercial contexts owing to heterogeneity in fruit quality (Johnson et al., 2013).

Date palms inhabit harsh desert environments and remain viable even in areas with saline soils and survive long periods with limited water supply (Nixon, 1951; Wickens, 1998; Maas and Grattan, 1999; Ramoliya and Pandey, 2003; Sané et al., 2005; Elshibli et al., 2016; Müller et al., 2017). Despite high tolerance to abiotic stress, date palms require large volumes of water to produce commercial grade fruit and suffer from lower productivity and reduced fruit quality when subject to drought and salinity stress (Alhammadi and Kurup, 2012; Hussain et al., 2012). Date palms can grow in soils up to 12 dS m^–1^ without showing symptoms of salt stress (Ramoliya and Pandey, 2003). However, Maas and Grattan (1999) reported that for every unit of increasing salinity above 4 dS m^–1^, date palm experience a 3.6% decline in yield. Therefore, modest increases to soil salinity can have measurable impacts on crop productivity (Hussain et al., 2012) and long term irrigation with saline water may not be commercially viable (Tripler et al., 2007, 2011). In addition to impacts on yield, salinity impacts important agronomic traits including extending the juvenile stage by 2 years and delaying fruit development in adults (Tripler et al., 2007, 2011).

Many date palm growing areas are increasingly affected by saline soils (Pitman and Läuchli, 2004; Malash et al., 2008; Haj-Amor et al., 2016), drought (Elshibli et al., 2016), falling water tables (see references in Al-Muaini et al., 2019a), and increased groundwater salinity (Alfarrah and Walraevens, 2018). These factors have had significant effects on date palm cultivation. For example, in the United Arab Emirates (U.A.E.), soil salinity is high in many areas due to over-irrigation with increasingly saline water such that a large percentage of farms have soil salinities in the 16–20 dS m^–1^ range (Dakheel, 2003). This has resulted in declining productivity in salt-affected areas and the abandonment of farms and crop failure in severe cases (Dakheel, 2003). In addition to regional concerns about date palm productivity, the high water usage requirements of date palm (i.e., up to 210 L per day per tree in the summer and approximately 1/3 of total groundwater use in the U.A.E.; Al-Muaini et al., 2019a) are putting pressure on regional authorities to moderate irrigation practices and evaluate the impact of irrigating date palms with increasingly saline water (Al-Muaini et al., 2019a, b). These concerns motivate expanding research on abiotic stress in date palm and laying a foundation for crop improvement.

Studies of date palm have begun to elucidate the mechanistic basis for abiotic stress tolerance in this species. For example, a recent report characterized a unique form of embryonic dormancy known as remote germination that protects organs and meristematic cells of early stage seedlings from dry surface soils and heat stress and may represent an adaptation to harsh desert conditions (Xiao et al., 2019). In the last few years, a number of studies have reported variation in the response to abiotic stresses among cultivars and their seedling progeny (Alhammadi and Edward, 2009; Al Kharusi et al., 2017; Al-Khateeb et al., 2019). Others have begun to probe the complex responses to abiotic stresses using genome-wide omic technologies such as transcriptomics, proteomics, methylomics, and metabolomics (Yaish and Kumar, 2015; Safronov et al., 2017; Yaish et al., 2017; Al-Harrasi et al., 2018; Rikek et al., 2019). Other recent developments include advances in transformation strategies for genetic engineering and gene editing (Prieto, 2011; Cardi et al., 2017), use of heterologous expression systems for studies of gene function (Patankar et al., 2019a, b), improvements to the date palm genome (Hazzouri et al., 2019), and demonstration of genome-wide association studies (GWAS) for mapping phenotypic trait variation in date palm (Hazzouri et al., 2019). Despite these advances, there is presently limited prospect of crop improvement using conventional breeding or genomic selection owing to many challenges faced by perennial fruit crops (Laurens et al., 2012).

Salt and drought stress response mechanisms in date palms and other plants has been reviewed in a number of recent comprehensive treatments (date palms: Alhammadi and Kurup, 2012; Hussain et al., 2012; plants: Munns and Tester, 2008; Hanin et al., 2016; He et al., 2019). In this review, we highlight recent work on the effects of salt and drought on *P. dactylifera* including studies using omics-based technologies and those examining root and soil microbiomes effects on stress tolerance in this species. We also provide a perspective on directions for future genomic research and emphasize a need for application of forward genetic approaches (e.g., GWAS) to complement genome editing and other reverse genetic approaches to dissecting the molecular basis of stress tolerance traits. Finally, we discuss the need for improved resources including well-established protocols for transformation and gene editing, plant materials such as those required for mapping studies, and broader application of omic-related technologies to diverse cultivars of *P. dactylifera* and its *Phoenix* wild relatives.

## Salinity

### Salinity Tolerance in Date Palms

Salinity induces detrimental changes to the anatomy, physiology, and growth of plants. These changes are counteracted by mechanisms that mitigate the effects of stresses including osmotic and ion toxicity stress. Salinity inhibits water uptake and increases the concentration of toxic ions such as Na^+^, threatens membrane integrity, results in the accumulation of reactive oxygen species (ROS), and contributes to imbalances in nutrient uptake. These effects have negative impacts on plant physiology including reduced photosynthetic capacity, impaired signaling, and alterations to cellular metabolism (Munns and Tester, 2008). These effects jointly contribute to reduced growth rates, increased rates of senescence, and lower yields of crops (Fricke et al., 2006; Sahi et al., 2006; Munns and Tester, 2008).

Plants mitigate these effects with diverse and complex sensing, signaling, and response pathways that determine the tolerance of a plant to salt (Hanin et al., 2016). These pathways interact to mount salt tolerance responses such as the production of compatible solutes (Munns and Tester, 2008), ion compartmentalization (e.g., in vacuoles), and neutralization of ROS. For example, plants neutralize ROS by producing antioxidant metabolites such as ascorbate, glutathione, and tocopherols or by expressing ROS-detoxifying enzymes such as superoxide dismutase (SOD), Ascorbate peroxidase isoenzymes (APX), and catalase (CAT) (Huang et al., 2019). Another strategy is to exclude salt ions from entering the root or restricting the transport of Na^+^ within the plant. These mechanisms include changes to root anatomy such as limiting xylem Na^+^ loading and translocation, modifying membrane permeability to exclude toxic ions, and active exclusion of ions from cells via ion pumps (Zhu, 2003; Munns and Tester, 2008; Hanin et al., 2016).

Many early studies characterized the impact of salinity on date palm growth, physiology and tolerance response and revealed that date palm respond to salt with many of the same strategies as other plants (Alhammadi and Kurup, 2012; Hussain et al., 2012). For example, salt stress triggers the production of osmolytes and compatible solutes in date palm including proline (Djibril et al., 2005; Yaish, 2015). Other studies have reported that date palm ameliorate the effects of ROS by increasing the expression of anti-oxidant enzymes (Ait-El-Mokhtar et al., 2019) and increasing concentrations of anti-oxidant metabolites (Al Kharusi et al., 2019a).

Recent studies have characterized the effects of salt on different cultivars (or their seedling progeny). For example, in a study of seedling progeny of 10 cultivars, Al Kharusi et al. (2017) suggested that date palms could be separated into salt tolerant and sensitive cultivars based on root and shoot growth characteristics. They reported that the most salt sensitive varieties have elevated Na^+^ in roots and shoots, reduced shoot K^+^, reduced relative water content in leaves, and higher electrolyte leakage. A similar study of seedling offspring from 12 date palm cultivars reported similar changes in Na^+^ and K^+^, but also reported decreased Ca^+^ and Mg^+^ particularly in roots and changes in nitrogen and phosphorus contents in both roots and shoots in response to salt treatments (Alhammadi and Edward, 2009).

Al Kharusi et al. (2019b) studied the seedling offspring of a salt tolerant variety, ‘Umsila,’ and salt sensitive variety, ‘Zabad.’ They reported that salt tolerant seedlings responded to salinity by developing a thicker protective Casparian strip in roots, increasing osmolyte and compatible solute concentrations including proline, glycine betaine, and total sugar. These changes were associated with increased photosynthesis rates and development of a larger root system and leaf areas. Another study by these same authors suggested that the seedling offspring of the salt tolerant variety also balance their uptake of Na^+^ and K^+^ and maintain a higher concentration of antioxidant metabolites (Al Kharusi et al., 2019a).

Two recent studies by Patankar et al. (2019a, b) have studied the function of date palm salinity-response genes by expressing them in heterologous systems. Metallothioneins (MTs) are cysteine rich proteins that play a role in reducing oxidative damage under abiotic stress conditions. Patankar et al. (2019a) expressed the date palm metallothionein gene, PdMT2A, in a salt-sensitive yeast (*Saccharomyces cerevisiae*) mutant which conferred tolerance to salinity, drought and oxidative stresses. Overexpression of PdMT2A in transgenic Arabidopsis resulted in reduced Na^+^ accumulation and maintenance of potassium/sodium (K^+^/Na^+^) ratio compared to wild type, which they attributed to the HKT transporter. In addition, transgenic lines showed higher chlorophyll content, higher superoxide dismutase activity (SOD) and better scavenging ability of ROS and were drought and oxidative stress tolerant.

In a related study, Patankar et al. (2019b) expressed date palm aquaporin PdPIP1;2 in yeast and reported improved tolerance to salinity and oxidative stresses. On the other hand, overexpression of the same gene in Arabidopsis showed symptoms of improved tolerance including enhanced biomass, chlorophyll content, root length under salt and drought conditions and high K^+^/Na^+^ compared to wild type. These two studies illustrate an approach to studying date palm gene function including the mechanisms by which genes may confer tolerance to abiotic stress.

These recent advances have provided new insight into the physiological basis for differences in the salinity response among date palm cultivars and highlighted approaches to characterizing the function of individual genes in salinity response pathways. At present, however, the genetic basis for variation in this response remains poorly understood and no candidate genes or mutations have been identified that might control variation in these traits among cultivars.

### Omic Studies of Salinity Tolerance in Date Palm

Omic technologies have the potential to yield a system-level perspective on salinity response mechanisms through characterization of stress inducible genes, regulatory networks and biochemical pathways. A number of studies have adopted NGS (Next Generation Sequencing)-based profiling of the transcriptome and methylome. Others have adopted metabolomics or proteomic approaches to characterize the salinity response (Table 1). For example, Radwan et al. (2015) compared salt-treated and control samples of ‘Deglet Beida’ seedlings and reported differential gene expression (DGE) of a large percentage of genes in young roots including downregulation of sodium uptake and transport genes and upregulation of the ABA-signaling pathway. Other differentially expressed genes included members of the cell wall suberization and DNA repair pathways, and a putative cinnamoyl reductase enzyme that may divert flux from the phenylpropanoid pathway into lignin biosynthesis necessary for strengthening the cell wall. Future studies might use single-cell transcriptome analyses (Liu and Trapnell, 2016) to evaluate these hypotheses (Libault et al., 2017; Palovaara et al., 2017; Shulse et al., 2019).

**TABLE 1:** Omic studies of abiotic stress in date palm.

Technology	Abiotic stress	Description	Date palm material	Publication
RNA-seq	Salinity	DGE^a^ analysis of root tissue from ‘Deglet Beida’ after salinity stress	Seedlings	Radwan et al., 2015
RNA-seq	Salinity	DGE analysis of leaf and root from ‘Khalas’ after salinity stress	Seedlings	Yaish et al., 2017
Small RNA-seq	Salinity	miRNA target assessment and DGE analysis of leaf and root from ‘Khalas’ after salinity stress	Seedlings	Yaish et al., 2015b
RNA-seq	ABA-treatment	Leaves were treated with ABA followed by DGE analysis between treatment and control	Seedlings	Müller et al., 2017
RNA-seq and methylomics	Salinity	Differential methylome and transcriptome analysis of ‘Khalas’ roots in response to salinity	Seedlings	Al-Harrasi et al., 2018
Proteomics	Drought and salinity	Proteomic analysis of 18-month palms subjected to drought and salinity stress	Tissue culture	Rabey et al., 2016
Metabolomics	Salinity and silicon treatments	Non-targeted metabolomics analysis on leaf and root tissues after treatments with salt and silicon.	Seedlings	Jana et al., 2019
RNAseq + Metabolomics	Mild heat, drought, and combined heat and drought	Transcriptomic and metabolomic analysis of *P. dactylifera* under mild heat, drought, and combined.	Seedlings	Safronov et al., 2017

*^a^DGE, differential gene expression.*

Another RNA-seq study reported DGE in the leaves and roots of salt-treated versus control samples (Yaish et al., 2017). Genes that were differentially expressed in the leaves had roles in photosynthesis, starch and sucrose metabolism, and oxidative phosphorylation, while differentially expressed genes in the roots function in tryptophan, purine, and thiamine metabolism. Some genes, including High-Affinity Potassium Transporter 8 (HKT8 = HKT1;5), vacuolar proton pump, and the auxin-conjugating enzyme GH3, were differentially expressed in both the leaves and the roots. The authors also observed the upregulation of phosphoenolpyruvate carboxylase (PEPC) in leaf in response to salt which they speculated could indicate salinity-induced activation of C4 or CAM photosynthesis pathways.

Yaish et al. (2015b) quantified microRNAs, or miRNA, expression in the leaves and roots of seedlings in control versus salt stressed conditions. They reported 57 and 25 miRNAs that were differentially expressed in leaf and root in response to salt stress. The authors listed a number of mRNA targets of these miRNAs that they speculated may be salinity-related including hormone response elements (e.g., abscisic acid responsive elements-binding factor), kinases, transcription factors, and transporters (Yaish and Kumar, 2015). The observation that microRNAs are differentially expressed in response to salinity stress suggests a possible role for these genes in the salinity response, although the authors cautioned that their observations would benefit from further validation.

Another study examined the effect of silicon on salt-stressed date palm seedlings. Jana et al. (2019) used metabolic profiling to measure thousands of metabolites, such as antioxidant compounds (e.g., pyridoxine, cepharanthine), osmoregulators (e.g., mucic acid) and intermediate detoxification (e.g., S-D-lactoylglutathione, beta-cyano-L-alanine) in roots and leaves that accumulated in response to silicon, salt, and in combination. They showed that in non-stressful conditions, silicon promotes growth of date palm seedlings, whereas in the salinity treatment, silicon acted as a negative regulator of salt stress. Studies of the differential accumulation of metabolites in response to silicon and salt treatments could yield insight into the protective role of silicon under salinity stress.

## Drought

### Tolerance to Drought in Date Palms

The availability of water is central to virtually all components of plant physiology and plants have evolved a complex array of mechanisms to maintain high water potential in drought conditions (Jarvis and Jarvis, 2006). Maintenance of water potential is achieved by minimizing water loss via transpiration and maximizing water uptake and includes rapid response mechanisms including closure of stomata (Cowan, 1977) and longer term changes to plant anatomy and gene expression (Chaves et al., 2003). For example, plants control water loss by modifying leaf characteristics, such as the production of cuticle wax (Hadley and Smith, 2011) and reducing leaf area and stomatal conductance through leaf senescence (Munné-Bosch and Alegre, 2004). At the physiological level, reduced water availability causes systemic changes in plant physiology including increased osmotic stress, reduced photosynthetic rates and the production of ROS. Among the most important responses to these effects are the production of molecular chaperones, anti-oxidants and compatible solutes via many of the same stress responsive pathways induced by salinity stress that constitute a general response to abiotic stress (He et al., 2018).

Date palms have a number of anatomical characteristics that contribute to tolerance of hyper-arid conditions. For example, date palms maintain thick, waxy cuticle and pinnately compound leaves covered with many spines, which insulate the tip growing point. The deep root system in date palms traps water in various types of soils. Those traits reduce evaporation and maximize water uptake and contribute to *P. dactylifera* tolerance for drought stress (Nixon, 1951; Wickens, 1998; Ramoliya and Pandey, 2003; Sané et al., 2005). Nevertheless, long periods of drought negatively impact date palm by reducing growth, fruit quality and yield (Elshibli et al., 2016).

One active area of research has focused on anatomical features of date palm roots that may represent adaptations to desert conditions including drought. Xiao et al. (2019) reported a novel form of germination where organs and meristem cells experience a period of developmental arrest. They reported that date palm develop a tuber-like structure called the cotyledonary petiole that protects the developing embryo in the soil. This same study also reported that date palms maintain suberized and lignified xylem, phloem and bundle cells in roots and produce pneumatophores, a specialized type of root. These authors speculated that these anatomical features may account for the adaptation of date palm to drought and salinity.

Arab et al. (2016), subjected 2 year old date palm seedlings to drought and heat. Briefly, the authors reported that photosynthesis was not affected by these stresses, despite a drop in the concentration of antioxidants including ascorbate and glutathione in leaves. The authors suggested that reduced concentrations of anti-oxidants may be compensated by a concomitant increase in the activity of a anti-oxidant enzyme, glutathione reductase. Furthermore, increased emission of isoprene under heat supported its role as an antioxidant. Finally, they also reported a change in fatty acid composition under drought, but not heat, which could suggest that date palms have independent response pathways to drought and heat stress.

A recent study by Yaish (2015) reported that date palm seedlings accumulate proline not only in response to drought and salinity stress, but also in response to extreme temperatures and abscisic acid treatments. They concluded that proline production is a common response for multiple stressors, which make it a possible marker in date palm breeding programs that aim to improve drought and salt tolerance.

In contrast to the study of salinity, fewer studies have assessed variation in the response of date palm cultivars to reductions in the availability of water. A recent study by Al-Khateeb et al. (2019) simulated osmotic stress in micropropagated date palm plantlets by adding mannitol to the culture medium. The three cultivars studied showed reduced root, shoot, and total biomass, intercellular CO_2_ assimilation rate, transpiration rate and water content in water-stressed conditions at the seedling stage. However, they reported that there were only weak differences among cultivars in their tolerances.

### Omic Studies of Water Shortage in Date Palm

There are presently few omics studies of drought in date palm (Table 1). A recent proteomics study identified genes involved in salt and drought tolerance in *P. dactylifera* (Rabey et al., 2016). The researchers challenged 3-month seedlings of the ‘Sagae’ cultivar with polyethylene glycol (82.5 g/L) and salinity (43 g/L) and identified 47 differentially expressed genes in the leaves. Thirteen of the genes were responsive to both salt and drought, 17 others were responsive only to salt stress, while the remaining only under drought. Some of the differentially expressed genes that were downregulated under drought included ribulose-1,5-bisphosphate, carboxylase/oxygenase, oxygen-evolving enhancer protein 2, chloroplastic-like, and cytochrome P450 implying the deactivation of the photosynthetic pathways in response to the treatment conditions.

Safronov et al. (2017) used transcriptomic and metabolomic profiling to characterize the response to heat and drought stress in *P. dactylifera* (Table 1). The two stresses had similar effects including the upregulation of soluble carbohydrates and increased antioxidant activity in the cytosol, chloroplasts, and peroxisomes. Differentially expressed genes involved in circadian and diurnal rhythm in response to combined heat and drought were reported and implied a novel stress-avoidance strategy.

Another study applied ABA to date palm leaves to mimick the effects of drought (Table 1; Müller et al., 2017). The authors reported a DGE analysis between ABA-treatment and control conditions and reported a broad overlap in differentially expressions genes in date palm and drought stress-responsive genes in Arabidopsis. For example, the date palm response to ABA includes well-known genes in Arabidopsis including phosphatases in the PP2C family, ATP binding cassette (ABC) transporters, late embryogenesis abundant proteins (LEAs) and MYB74, a guard cell transcription factor.

## The Date Palm Microbiome and Abiotic Stress

Plants have evolved associations between roots and soil microbes that confer tolerance to abiotic stress. This root-associated microbiome, or rhizobiome, consists of plant roots and their associated bacteria and fungi that alter plant development and physiology, confer resistance to pathogens, and confer tolerance to various abiotic stresses such as salinity and drought (Mefteh et al., 2017; Yaish et al., 2017; Jones et al., 2019). For example, plants improve their tolerance of abiotic stress by altering root exudates and modifying the species composition of the rhizobiome (Berg et al., 2013; Mapelli et al., 2013; Hacquard et al., 2017; Berendsen et al., 2018; Sasse et al., 2018; Whitaker et al., 2018). The microbiome enhances stress resistance by promoting osmolyte accumulation, alleviating oxidative stress by enzymatic and non-enzymatic mechanisms, or synthesizing hormone-like substances that modulate root expansion and hormone homeostasis (Bérard et al., 2011; De Zélicourt et al., 2013).

The ability of the plant root system to be colonized by endophytes (beneficial microbes able to colonize the root inner tissues) is essential for plants to receive benefits including protection against abiotic stress. For instance, bacterial acetyl co-carboxylase deaminase (ACCD) enzyme facilitates plant growth under environmental constraints and was found to help endophytic colonization within plants (Sessitsch et al., 2012). Heterologous expression of ACCD in *P. dactylifera* could promote the colonization of various beneficial endophytes.

*Phoenix dactylifera* thrives in oasis ecosystems, where microbial communities help plants to tolerate environmental extremes (Kumar et al., 2011). Although endophytic bacteria enhance plant growth under abiotic stress (Rolli et al., 2014), there are few studies on *P. dactylifera* endophytic bacteria and their role in the acquisition of salt and drought tolerance. The use of NGS will enhance the characterization of endophytic microbiota of *P. dactylifera* and would lead to a better understanding of the biodiversity in the rhizosphere. This would help dissect the function of beneficial microbial symbiosis and the molecular mechanisms by which symbiosis is established and exerts beneficial effects (Köberl et al., 2011; Marasco et al., 2012).

The root and leaf microbiomes of date palm represent diverse communities comprised of bacterial and fungal species. In a recent study in the Sahara Desert in Tunisia, the bacterial communities selected by the root system of date palm were dominated mainly by Gammaproteobacteria and Alphaproteobacteria irrespective of the edaphic conditions or geographical location (Mosqueira et al., 2019). A study by Cherif et al. (2015) of the ecology of date palm root endophytes from oasis desert farms in southern Tunisia indicates that date palm roots select diverse endophytic communities that are able to promote plant growth under drought conditions. Another study identified endophytic bacterial and fungal communities in *P. dactylifera* grown under salt stress using pyrosequencing and showed that the composition of those microbial communities changed significantly in response to changes in salinity (Yaish et al., 2015a, 2016).

The most common fungal endophytes isolated from *P. dactylifera* are *Penicillium citrinum* isolate TDPEF34 and *Geotrichum candidum* isolate TDPEF20, which represent a promising source of diverse bioactive metabolites (Mefteh et al., 2018). The most frequently isolated genus of endophytic bacteria from *P. dactylifera* is Pseudomonas, which is well-known for its growth-promoting properties (Roca et al., 2012; Skz et al., 2013). In drought-like conditions, innoculation of date palm roots with these endophytic bacteria promotes growth (Cherif et al., 2015). Pseudomonas isolated from *P. dactylifera* showed a number of potential plant growth promoting (PGP) properties including enhanced inorganic phosphate solubilization, nitrogen fixation, and the production of siderophores, phytohormones,1-aminocyclopropane-1-carboxylate deaminase, and exopolysaccharide.

Fungi also provide PGP services. The most studied group of PGP fungi are the Arbuscular Mycorrhizal Fungi (AMF), which belong to the Glomeromycota and form symbiotic associations with plants by colonizing the root. A recent study of the effect of innoculating date palm roots with AMF improved tolerance under drought and salt-stressed conditions (Meddich et al., 2018). Ait-El-Mokhtar et al. (2019) also reported that date palm seedling roots colonized by AMF structures improved tolerance to salt stress.

The research focus has changed in the past few years from the identification of individual microbial strains with growth-promoting effects to metagenomic studies of the abundance and diversity of root microbiomes. Studies that have applied high-throughput sequencing analyses have revealed that the rhizosphere niche is an ecological hotspot where roots host a tremendous array of microbial taxa (Bulgarelli et al., 2013; Busby et al., 2017; Yu et al., 2018; Khare et al., 2018). NGS-based technologies have yet to be applied to studies of the date palm root microbriome.

There are many strategies for engineering the plant microbiome such as host-mediated and multi-generation microbiome selection, inoculation of bulk soils and the rhizosphere, and other approaches (Orozco-Mosqueda et al., 2018; Timm et al., 2018; Jochum et al., 2019a, b; Khan et al., 2019). Engineering of the root-associated microbiome can be used to alter microbiome composition and potentially improve tolerance to abiotic stress. While bioengineering of the plant microbiome is in its infancy, it is an interesting option to improve the biological capabilities of plants (Qiu et al., 2019).

## Perspectives

Increases in ground and soil salinity and depletion of fresh water resources necessitate characterization of abiotic stress response pathways and creation of a road map that outlines steps toward developing a more tolerant date palm crop. Breeding for improvement in *P. dactylifera* using conventional breeding was conducted in the United States into the 1970s but has since been terminated (Krueger, 2001). The prospect for improvement via conventional breeding or modern approaches, such as genomics-assisted breeding (Kole et al., 2015) or genomic selection as used in oil palm (Nyouma et al., 2019), faces challenges owing to significant economic and technical constraints. However, despite many challenges, it is our belief that a combination of omics, forward genetics, and reverse genetics approaches provide a potential path to improvement of the data palm crop. Below we describe approaches to genetic mapping of stress tolerance traits that can yield candidate genes and mutations that control variation in tolerance among cultivars. We then describe how genes discovered by genetic mapping can be targeted by gene editing [e.g., by clustered regularly interspaced short palindromic repeats (CRISPR) associated protein 9 (Cas9)] toward the goal of engineering varieties with improved stress tolerance.

### Genome Resources

There is a need for continued improvement to the genome assembly and gene annotation of date palm. There are presently three draft assemblies including two female (Al-Dous et al., 2011; Al-Mssallem et al., 2013) and one male genome (Hazzouri et al., 2019). The two female draft genomes are fragmented assemblies of the ‘Khalas’ cultivar with low contiguity, while the male assembly is derived from a fourth generation backcross male of a cross with the ‘Barhee’ cultivar as the recurrent parent. This BC4 male assembly is the only one of the three genomes to include long read sequencing technology (i.e., Pacific Biosciences), integrate a genetic map to place contigs on linkage groups (Mathew et al., 2014), and use a diploid aware assembler (i.e., FALCON-Unzip). The BC4 male primary assembly represents a substantial improvement to previous assemblies that consists of approximately 50% of the genome sequence being placed on the 18 linkage groups (Hazzouri et al., 2019). The primary sequence and gene models can be accessed at the Date Palm Genome Hub website^1^ (Hazzouri et al., 2019).

There remains much room for improvement of the date palm genome. Some of the factors currently limiting improvement are the absence of a high density genetic map and the heterozygosity of date palm cultivars including the BC4 male. Improvements to the current assemblies can be achieved by adopting improved diploid aware assembly software, inclusion of a high density genetic map, and incorporation of additional technologies (e.g., Hi-C, Dudchenko et al., 2017). Despite the prospect of additional improvements using these methods, a high quality assembly for date palm may require generation of a homozygous double haploid variety (Das et al., 2018). For example, sequencing of a double haploid contributed to dramatic improvements to the apple genome compared with an earlier assembly derived from a heterozygous sample (Daccord et al., 2017). Improvements to the date palm genome will assist in many areas of abiotic stress research including providing a more complete set of gene models and chromosome-level sequences that will improve the prospect of discovering candidate genes with genetic mapping and enhance discovery using many other omic technologies.

### Linkage Mapping and GWAS

A primary objective of mapping studies is to determine loci that control heritable variation in phenotypic traits. These ‘forward genetic’ approaches comprise a powerful set of methods to identify genes that control variation in phenotypic traits and dissect their genetic basis. Genetic mapping can yield candidate genes and mutations that control variation in a trait and suggest strategies for its modification using genetic engineering. In other cases, genetic mapping can lead to discovery of linked markers that can be used in marker-assisted selection (MAS) (Das et al., 2017) and crop breeding.

Linkage mapping of QTL in fruit and other tree crops is typically initiated with a cross between non-inbred parents (Khan and Korban, 2012). In the simplest experimental design, full-sib progeny of outbred parents are genotyped at set of anonymous markers and phenotyped for a trait of interest. Linkage mapping is then conducted to identify marker-trait associations using statistical models appropriate for this design (e.g., the double pseudo-testcross approach; Grattapaglia and Sederoff, 1994). This approach has been applied to map naturally occuring variants in many forest trees and fruit crops (Wu et al., 2010; Khan and Korban, 2012) and could in principle be applied to map traits in plants produced by mutagenesis of somatic embryogenic cell suspensions (Jain, 2012). Linkage mapping is possible in date palm as hundreds to thousands of seedlings from controlled crosses (i.e., pollen from a single male used to pollinate a single female) can be generated to produce full-sib progeny for standard linkage mapping or half-sib progeny (i.e., one male used to pollinate different female cultivars) for use in a pedigree-based mapping designs.

An alternative approach is to leverage natural variation to map traits using GWAS (Khan and Korban, 2012; Korte and Farlow, 2013). In a typical GWAS experiment, hundreds of unrelated samples are phenotyped typically in a common garden such as a nursery or farm for a trait of interest and then genotyped (e.g., using NGS-based whole genome re-sequencing). This is an attractive alternative with a number of advantages over linkage mapping. First, a trait is more likely to segregate in a large GWAS panel than in a cross between two samples. Second, QTL intervals are smaller in GWAS studies in most tree and fruit crops due to the rapid decay of linkage disequilibrium (Khan and Korban, 2012). This makes discovery of candidate genes more likely provided that marker density is sufficiently high to detect marker-trait associations.

Mapping approaches have not been widely applied in date palms. Hazzouri et al. (2019) conducted GWAS on fruit-related traits in 145 varieties of date palm using high density genotyping using moderate coverage (i.e., the average number of sequencing reads spanning each genomic position) whole genome Illumina sequencing reads mapped to an improved genome assembly. In date palm, the decay of linkage disequilibrium is sufficiently fast (Hazzouri et al., 2015; Flowers et al., 2019) that GWAS yielded candidate genes and probable causal mutations for fruit color and fruit sugar composition (Hazzouri et al., 2019). The successful high resolution mapping of these traits demonstrated the viability of combined NGS-based sequencing and standard structured association mapping in date palm and produced markers for sex determination and commercially important fruit traits that could in principle be incorporated in future MAS experiments.

**FIGURE 1:**
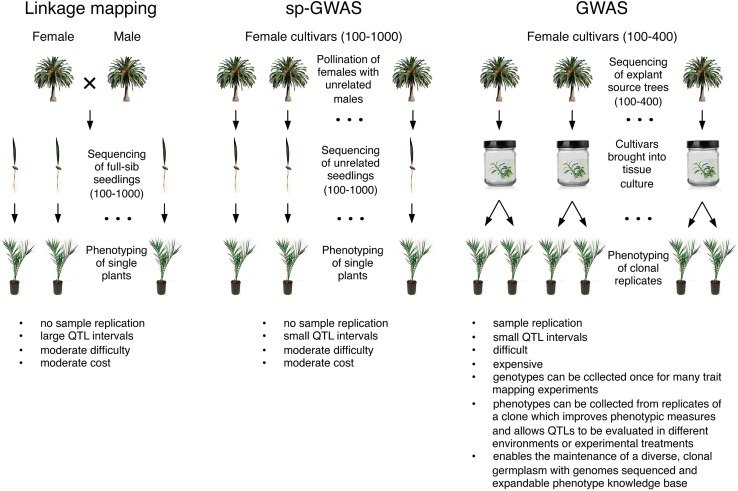
Approaches to mapping abiotic stress traits in date palm.

Mapping abiotic stress-related traits in adult date palms is currently intractable owing to the long juvenile stage and cost of growing and maintaining large mapping populations. An alternate solution would be to map such traits in early stage seedlings where environmental conditions can be carefully controlled. For example, linkage mapping on full-sibs from a controlled cross, GWAS on unrelated seedlings (e.g., diverse female cultivars pollinated with unrelated males), or a hybrid approach such as F1 association mapping (FOAM, Romero Navarro et al., 2017) are all plausible approaches to mapping in early stage date palm seedlings. A disadvantage of these approaches is that individual seedlings cannot be cloned easily for the purpose of generating replicate samples for phenotyping owing to the difficulty of establishing new micropropagation lines (Mazri and Meziani, 2015). These approaches therefore require phenotyping single samples as is common in animal and human genetics, but less common in plants. Such single plant linkage mapping or GWAS (“sp-GWAS,” Gyawali et al., 2019) suffers from increased error in phenotypic measurements owing to lack of replication and the inability to phenotype a seedling genotype in multiple environments or treatments (Figure 1).

An alternate approach would be to conduct GWAS on early stage plants from diverse varieties propagated in tissue culture (Figure 1). Use of micropropagated varieties would (1) allow expensive genotyping steps to be performed only once on a clonal lineage followed by phenotyping of many traits, (2) facilitate phenotyping of replicates of a clone thereby reducing the effects of measurement error and plant-to-plant variability in phenotypic measures, (3) allow experiments to be replicated in different environments or treatments to ensure stability of QTLs (4) allow more complex experimental designs to be adopted. For example, clones of each genotype would allow paired measurements of phenotypic responses to abiotic stress through measurements of the phenotype in a clone grown in both treatment and control conditions. Adoption of tissue culture-propagated cultivars in large-scale experimental programs would require a significant effort including expansion of the numbers of cultivars currently being propagated in tissue culture.

Mapping of early stage stress response traits from micropropagated cultivars or seedling offspring from controlled crosses suffers from additional challenges. First, sample sizes required for successful mapping depend on the genetic architecture of the trait. Even the simplest traits can require hundreds of samples and more complex traits may require much larger sample sizes. Second, both approaches require genotyping of large numbers of seedling progeny or clonally propagated varieties which in date palm are both highly heterozygous. NGS-based whole genome re-sequencing approaches to genotyping offer the best opportunity to identify candidate genes and mutations. However, use of this approach to genotype highly heterozygous samples likely requires moderate to deep sequencing because imputation – the use of linkage information to infer missing genotypes – may not be possible without reference panels and low coverage sequencing approaches used in inbred crops (Wang et al., 2016) may not be viable owing to high genotyping error rates at heterozygous sites. Reduced representation libraries [e.g., Genotype-by-sequencing (GBS); Elshire et al., 2011)] or array-based genotyping may provide more cost effective solutions, but they may yield insufficient marker density to map traits with GWAS (Figure 1).

### Genetic Transformation and Gene Editing

The genetic engineering of date palm lags behind that of other species such as rice, barley, and maize and fruit crops such as apple (Waltz, 2015; Nishitani et al., 2016) and oil palm (Budiani et al., 2018) where genetic transformation has been adopted for improvements at the commercial level. The factor currently limiting advancements in date palm is that there is not a well-established transformation protocol. Attempts to transform *P. dactylifera* using either *Agrobacterium tumefaciens* or microprojectile bombardment has been met with limited success, and no conclusive report of stable transformants of an expressed gene in date palm have yet been successful (Jain, 2012).

Genome editing methods, such as zinc finger nucleases (ZFNs), transcription activator-like effector nucleases (TALENs), and CRISPR/Cas9 have all enhanced the prospect of genetic modification of crops (Kanchiswamy et al., 2015). The former technologies are expensive and time consuming and require protein engineering, which makes them less suitable and limits their application (Jaganathan et al., 2018), while CRISPR/Cas9 is popular because it is affordable, scaleable, and relatively simple to apply (Jia and Wang, 2014; Jia et al., 2016).

CRISPR/Cas9 gene editing can be used to knockout, activate or repress the expression of target genes. Successful editing can use transformation of a construct containing Cas9 and a guide RNA (sgRNA) that is homologous to a target gene in the plant genome, or may use alternative “DNA-free” approaches such as CRISPR/Cas9 ribonucleoproteins (RNPs). Key considerations for the prospect of CRISPR/Cas9 editing in plants include the gene to target, the sgRNA sequence, the delivery method and plant tissue (e.g., callus), and the regeneration of fertile plants (Altpeter et al., 2016; Wang et al., 2019). Many of these and additional considerations are reviewed in detail by Sattar et al. (2017) in the context of improvement of date palm. For example Sattar et al. (2017) highlighted that high heterozygosity of date palm cultivars can make it challenging to design the sgRNA, which must match a target region near a protospacer adjacent motif (PAM) site.

CRISPR/Cas9 offers a number of benefits well-suited to modification of dioecious tree crops such as date palm (Kole et al., 2015). Any strategy for improvement in tree crops should prioritize development of a modified plant in as few generations as possible, ideally a single generation. One advantage of CRISPR/Cas9 in this respect is that it produces biallelic edits that result in homozygous changes at the target site, which eliminates the need for a genetic cross to produce homozygous alterations (Kole et al., 2015). Another consideration is the need to minimize the footprint of the gene editing procedure in the date palm genome. For example, transformation of somatic embryos with *Agrobacterium* can lead to random integration of bacterial plasmids which may contribute to GMO-related regulatory constraints (Sattar et al., 2017). A possible alternative is the use of next-generation DNA-free CRISPR/Cas9 RNPs, which can be delivered directly into protoplasts as has been done in apples in an effort to increase resistance to fire blight disease (Malnoy et al., 2016).

Lessons from genetic engineering of oil palm (*Elaeis guineensis*) may help with developing strategies for *P. dactylifera*. For example, both microprojectile bombardment (Kadir et al., 2015) and *Agrobacterium* (Budiani et al., 2018) have been used to transform oil palm. Moreover, Crispr/Cas9 technology has been succesful in oil palm. Budiani et al. (2018) used *Agrobacterium* to introduce the CRISPR/Cas9 constructs for editing isoflavone reductase and metallothionein -like protein in an effort to introduce resistance to *Ganoderma*. Given the success in other crops, we anticipate that CRISPR/Cas9 will soon provide a means for creating stable site-directed gene edits in date palm and may provide the best chance at modification of date palm.

### *Phoenix* Crop Wild Relatives

Another under-utilized resource in the study of abiotic stress in date palm is the wild relatives of date palm. Members of the genus *Phoenix* are known to occupy diverse habitats ranging from the banks of the Mekong River (*Phoenix roebelenii*) and coastal areas subject to salt-water incursion (*Phoenix theophrasti*) (Barrow, 1998). The range of habitats occupied by *Phoenix* wild relatives suggests that these species harbor a diversity of stress-tolerance traits that could be exploited for the study and improvement of date palm. Incorporation of wild relatives in experimental programs is becoming increasingly important in perennial crop improvement strategies (Migicovsky and Myles, 2017) and first steps toward characterizing this diversity have been taken through whole genome sequencing of the closest relatives of date palms (Gros-Balthazard et al., 2017; Flowers et al., 2019).

Exploitation of diversity in wild relatives of cultivated *Phoenix* may benefit from the fact that many species in this genus readily hybridize and produce viable hybrids either from seed generated from inter-specific crosses or from somatic embryogenesis (Gros-Balthazard, 2013). A prospective area for future research is to characterize differences in abiotic stress tolerance in the crop wild relatives of date palm. In the long term, it may also be possible to map traits in inter-specific crosses as has been done in oil palm (Osorio-Guarin et al., 2019) or transfer beneficial traits into date palm using somatic embryogenesis (Sudhersan et al., 2009).

## Conclusion

Application of omic methods has begun to detail the genes and biochemical pathways that control the response to abiotic stress in date palm. Many of these pathways such as the abscisic acid pathway are known from studies of other crops, but others including circadian and diurnal rhythm pathways may suggest novel pathways in date palm (Safronov et al., 2017). Future advances will benefit from combining omic approaches with reverse and forward genetics. For example, Yaish and Kumar (2015) previously advocated for reverse genetics approaches (e.g., site-directed mutagenesis and recombinant DNA technologies) to the study of abiotic stress in date palm. Indeed, development of a well-established protocol for transformation of callus or other micropropagated tissues and development of CRISPR/Cas9 or gene editing technology for date palm (Sattar et al., 2017) would present new opportunities for functional studies of abiotic stress tolerance and crop improvement. However, we also argue that genetic mapping offers a complementary set of methods that can be applied to identify QTLs that control variation among cultivars in traits such as abiotic stress resistance. Localization of QTLs to narrow genomic regions, when combined with RNA-seq and other functional omic data, can lead to discovery of candidate genes and causal mutations. In principle, candidate genes and mutations identified in this fashion could then be modified with gene editing techniques in stress sensitive elite commercial cultivars.

The prospect of improvement of date palm to abiotic stress described above is a long term goal. Achieving this goal will require development of a detailed road map with input from scientists from multiple disciplines and various stakeholders. However, a critical review of improvement for abiotic stress tolerance in cereals and other annual crops reported that attempts at improvement have had limited success. Some of the challenges include the multi-genic nature of stress resistance traits and QTLs for yield traits are often unstable across environments. For example, improved genotypes often show higher yields under stress, but lower yields in non-stressed conditions (Flowers, 2004).

For the near term, we suggest that efforts in date palm focus on stream-lining reverse genetic technologies including transformation methods and CRISPR/Cas9 gene editing, expanding germplasm resources (e.g., increased numbers of cultivars in tissue culture), GWAS mapping of abiotic stress traits, application of omic technologies to diverse cultivars and crop wild relatives, and characterization and manipulation of soil and root microbiomes. Achieving these goals would dramatically improve the outlook for crop improvement in date palm.

## Author Contributions

KA, KH, KM, and JF organized the preparation and wrote the manuscript. JF generated the figure. DN and AL assisted with writing and editing the manuscript.

## Conflict of Interest

The authors declare that the research was conducted in the absence of any commercial or financial relationships that could be construed as a potential conflict of interest.

## Footnotes

1 https://datepalmgenomehub.abudhabi.nyu.edu
